# A composite neonatal adverse outcome indicator using population-based data: an update

**DOI:** 10.23889/ijpds.v5i1.1337

**Published:** 2020-08-12

**Authors:** S Todd, J Bowen, I Ibiebele, J Patterson, S Torvaldsen, F Ford, M Nippita, J Morris, D Randall

**Affiliations:** 1 The University of Sydney Northern Clinical School, Women and Babies Research, St Leonards, NSW 2065, Australia; 2 Northern Sydney Local Health District, Kolling Institute, St Leonards, NSW 2065, Australia; 3 NSW Biostatistics Training Program, NSW Ministry of Health, St Leonards, NSW 2065, Australia; 4 Department of Neonatology, Royal North Shore Hospital, St Leonards, NSW 2065, Australia; 5 School of Public Health and Community Medicine, University of New South Wales, NSW 2033, Australia; 6 Department of Obstetrics and Gynaecology, Royal North Shore Hospital, St Leonards, NSW 2065, Australia

## Abstract

**Introduction:**

Severe morbidity rates in neonates can be estimated using diagnosis and procedure coding in linked routinely collected retrospective data as a cost-effective way to monitor quality and safety of perinatal services. Coding changes necessitate an update to the previously published composite neonatal adverse outcome indicator for identifying infants with severe or medically significant morbidity.

**Objectives:**

To update the neonatal adverse outcome indicator for identifying neonates with severe or medically significant morbidity, and to investigate the validity of the updated indicator.

**Methods:**

We audited diagnosis and procedure codes and used expert clinician input to update the components of the indicator. We used linked birth, hospital and death data for neonates born alive at 24 weeks or more in New South Wales, Australia (2002–2014) to describe the incidence of neonatal morbidity and assess the validity of the updated indicator.

**Results:**

The updated indicator included 28 diagnostic and procedure components. In our population of 1,194,681 live births, 5.44% neonates had some form of morbidity. The rate of morbidity was greater for higher-risk pregnancies and was lowest for those born at 39–40 weeks’ gestation. Incidence increased over the study period for overall neonatal morbidity, and for individual components: intravenous infusion, respiratory diagnoses, and non-invasive ventilation. Severe or medically significant neonatal morbidity was associated with double the risk of hospital readmission and 10 times the risk of death within the first year of life.

**Conclusion:**

The updated composite indicator has maintained concurrent and predictive validity and is a standardised, economic way to measure neonatal morbidity when using population-based data. Changes within individual components should be considered when examining longitudinal data.

## Introduction

Neonatal morbidity is a commonly used indicator of the quality of maternity care and neonatal wellbeing, particularly as rates of neonatal mortality are decreasing in countries with advanced healthcare systems [1, 2]. Our research group developed a composite indicator, the neonatal adverse outcome indicator (NAOI), in 2012 to retrospectively identify infants with severe or medically significant neonatal morbidity using selected diagnoses and procedures from Australian routinely-collected birth, hospital and death data [3]. Using such a composite indicator from population data is a cost-effective way to monitor safety and quality of perinatal services and investigate population health outcomes. Such indicators can be adapted and applied to the health systems of different countries in a standardised manner. To date, this indicator has been used not only by ourselves [4-7] but by other Australian [8] and international research groups [9-12].

Composite indicators overcome the issues associated with single diagnoses such as low predictability and poor ascertainment. For example, the 5-minute Apgar score, a measure that is commonly used to identify infants with poor birth outcomes, is a poor descriptor of neonatal morbidity [13]. Relying on specific diagnostic flags is problematic due to under-ascertainment, as has been previously shown for enterocolitis and intraventricular haemorrhage [14]. As severely unwell neonates are likely to have multiple diagnoses and/or procedures recorded, under-ascertainment of one diagnosis or procedure should be balanced by other components in a composite indicator. However, the inclusion of multiple components requires careful monitoring of individual component changes, such as changes in coding rules or clinical practice, which can undermine the integrity of the indicator. In Australia, all hospitals use the *International Statistical Classification of Diseases and Related Health Problems, Tenth Revision, Australian Modification* (ICD-10-AM) and the *Australian Classification of Health Interventions* (ACHI) for coding patient diagnoses and procedures respectively, with rules governed by the *Australian Coding Standards* (ACS) [15]. Updates to these standards occur approximately every two years to reflect changes in practice, technology use and diagnostic criteria.

Here we update the NAOI to reflect these changes, and re-evaluate the validity of this composite indicator by examining outcomes for infants in the year following birth using linked birth, hospital and death population-based datasets. This indicator is intended for retrospective investigation of morbidity burden and for comparison between different centres or regions, rather than for clinical use.

## Methods

### Refinement of existing NAOI

The development of the original composite indicator to select components reflecting severe or medically significant morbidity but not service utilisation is detailed elsewhere [3]. This initial indicator included 15 diagnosis and 7 procedure groups. These diagnosis and procedure groups followed the then current ICD-10-AM and the affiliated ACHI standards respectively (collectively ICD-10-AM/ACHI/ACS, 5^th^ edition) [16].

We reviewed all 22 diagnosis and procedure groups and updated them to the latest available standard (ICD-10-AM/ACHI/ACS, 10^th^ edition) [17]. Diagnosis and procedure codes were audited to identify which were still in use, had been updated, or had been superseded by new codes, using documentation provided by Australian Consortium for Classification Development [15]. Additionally, any new codes within a coding block that had multiple existing codes used in the NAOI were selected for review. These codes were combined to form a preliminary version of the updated indicator. For new codes an iterative process based on expert clinical and coding input was used to decide which to include in the updated indicator.

Our indicator defines severe neonatal morbidity as: (1) any record of specified diagnoses or procedures in the first hospital admission (birth admission); and/or (2) a record of death within the first 28 days of life. We avoided the inclusion of factors that reflect service provision, such as neonatal intensive care unit or special care nursery admission and length of stay, as these measures may differ between hospitals according to their neonatal service level. ‘Severe’ morbidity according to this definition can range from supporting (but medically significant) procedures such as non-invasive ventilation and intravenous infusion, to life threatening diagnoses such as septicaemia. However, all neonates identified by the NAOI would require specialised care because of severity of illness or need for a procedure (such as mechanical ventilation or intravenous infusion).

### Study population and data sources

The study population included all livebirths from 24 to 44 completed weeks’ gestation born to mothers residing in New South Wales (NSW), Australia from January 2002 to December 2014. NSW is the most populous state in Australia and approximately 96,000 births are recorded per year, representing 32% of all Australian births [18]. Data were taken from the NSW Perinatal Data Collection (birth data), formerly known as the Midwives Data Collection, and the NSW Admitted Patient Data Collection (hospital data). The NSW Perinatal Data Collection is a population-based surveillance system covering all live births and stillbirths in NSW of at least 20 weeks’ gestation or at least 400 grams birthweight. The NSW Admitted Patient Data Collection captures all inpatient separations (discharges, transfers and deaths) from all public and private hospitals in NSW. For each inpatient separation, diagnoses and procedures are coded from medical records by qualified clinical coders, with coding undertaken and recorded after the patient is discharged. The Registry of Births, Deaths and Marriages dataset (death data) was used to identify all in or out of hospital deaths within the first 28 days of life. These datasets were probabilistically linked by the Centre for Health Record Linkage. Linkage and analysis were approved by the New South Wales Population and Health Services Research Ethics Committee (2012/12/430) and only de-identified data were available for use by researchers.

### Data analysis

Data analysis was undertaken using SAS® Enterprise Guide® software, Version 6.1 (SAS Institute Inc., Cary, NC, USA).

The incidence of neonatal morbidity overall and for each of the diagnosis or procedure components was calculated for the entire cohort and for the first and last year of the study period (2002 and 2014). Cochran-Armitage trend tests were used to assess the change in incidence over time for each component, with significance set at P < 0.01. The influence of each individual component on the NAOI was determined by removing it from the indicator and calculating the incidence decrease. Gestational age-specific rates of neonatal morbidity were also calculated as an assessment of validity. Infants with missing hospital records were retained for these analyses, as they made up only a small proportion of the cohort (n = 8,676; 0.7%) and neonatal morbidity could still be identified by birth data indicator components alone. For records with no missing data, the odds of neonatal morbidity was reported for select maternal and infant characteristics. For infants discharged home, rates of hospital readmission (any and overnight) and death within the first year of life were reported.

## Results

The final indicator components are listed in Table 1. Changes from the original published version of the NAOI [3] are summarized in Box 1, and explanations for specific changes in Box 2. The majority of changes were due to the replacement of superseded procedure codes. The largest change in coding was for non-invasive mechanical ventilation, which migrated from coding individual procedures (such as continuous positive air pressure) to a combined ‘time spent on ventilation’ coding group, with time spent on invasive and non-invasive ventilation coded separately. Only one code (‘intravenous administration of an anti-infective agent’) was removed as it was determined that, in the absence of other indicators of morbidity (such as sepsis), administration may be for prophylaxis only and not an independent indicator of morbidity.

Box 1. **Summary of ICD-10-AM/ACHI/ACS coding changes in the neonatal adverse outcome indicator since previous publication [3].**

**Added:**

…because they replaced a previously used code

- Management of endotracheal intubation, single lumen: 22007-01, replaces 90179-03
- Management of endotracheal intubation, double lumen: 22008-01, replaces 90179-04
- Management of non-invasive ventilatory support: 92209, replaces 92038-00, 92039-00
- Insertion of feeding jejunostomy tube: 31462-00, replaces 30375-16
- Open tracheostomy, temporary: 41881-00, replaces 41883-00
- Open tracheostomy, permanent: 41881-01, replaces 41883-01
- Revision of tracheostomy: 41881-02, replaces 41883-02
- Therapeutic thoracentesis: 38803-00, replaces 38403-00
- Insertion of intercostal catheter for drainage: 38806-00, replaces 38409-00

…because they were new and/or within a coding block already in use, and indicative of serious morbidity

- Right heart catheterisation: 38200-00, within block 667
- Left heart catheterisation: 38203-00, within block 667
- Removal of lesion involving posterior cranial fossa: 90032-00, new code in 2008
- Closure of left atrial appendage: 96220-00, new code in 2015
- Percutaneous closure of left atrial appendage: 96219-00, new code in 2015

…because they were needed to create a consistent code history

- Injection of gamma globulin: 92181-00, consistent with 96199-05
- Injection of antidote: 92182-00, consistent with 96199-04
- Injection of insulin: 92183-00, consistent with 96199-06
- Injection of electrolyte: 92184-00, consistent with 96199-08
- Injection of anticoagulant: 92185-00, consistent with 96199-01
- Injection of steroid: 92188-00, consistent with 96199-03
- Parenteral infusion: 92192-00, consistent with 96199-07
- Intermittent positive pressure breathing [IPPB]: 92040-00, consistent with 92209

**Removed:**

- Intravenous administration, anti-infective agent: 96199-02 (see Box 2 for further details).

**Changed:**

- An upper limit of 999 hours was added to recorded hours of mechanical ventilation. Records with 1000+ hours had an increased probability of having no other morbidity flag, suggesting these values were errors.

Box 2. **Adaptations to refine specificity of neonatal adverse outcome indicator.**

**Removal of intravenous infusion of an anti-infective agent**

A large number of records were flagged only with ICD code 96199-02 (intravenous administration, anti-infective agent). As detailed in ACS coding standard 1617 (*Neonatal sepsis/risk of sepsis*), prophylactic antibiotics or antibiotics for ‘risk of sepsis’ are given to neonates if certain risk factors are met, even if no symptoms are seen [17]. Risk factors include preterm labour, premature rupture of membranes, signs of maternal infection, multiple birth with delay in delivery of subsequent infants(s), prolonged rupture of membranes, maternal carriage of group B streptococcus infection, or previous baby with invasive group B streptococcus disease. This suggests that, in the absence of other morbidity indicators (e.g. sepsis, respiratory conditions), intravenous infusion of anti-infective agents alone is a poor indicator of severe neonatal morbidity, so this component was removed.

**Refinement of non-invasive mechanical ventilation** In 2008 (6^th^ edition) non-invasive mechanical ventilation underwent a major revision, transferring from procedure-based (e.g. continuous positive airway pressure, intermittent positive pressure breathing, intermittent positive pressure ventilation, bi-level positive airway pressure subheadings) to time-based coding (management of non-invasive ventilatory support ≤24 hours, >24 – <96 hours, 96+ hours). As a consequence, this coding group 92209 now includes previously unrecorded procedures (e.g. high flow nasal cannula). While we have added the coding group 92040-00 (intermittent positive pressure ventilation) to create a more consistent population over time, it should be emphasised that the post-2008 non-invasive mechanical ventilation indicator component is a different population than pre-2008, containing the less serious face mask or nasal mask/prong/tube ventilation procedures. This information is detailed in ACS coding standard 1006 [17].

The inclusion of this specific NAOI component should be performed with consideration by the researcher, given that it may be a less sensitive measure of morbidity. Caution is advised when using this component across 2008 (**see Supplementary Figure 1**).

**Table 1: Diagnosis and procedure indicator components for the updated neonatal adverse outcome indicator (NAOI).** * A birthweight of < 2500g was also required to account for potential reporting errors. † This is a numeric field recorded in the hospital data separate from ACHI mechanical ventilation procedure codes ‡ The addition of this coding group to the NAOI should be considered according to the time period of the study. Longitudinal studies, particularly those crossing 2008, should be undertaken with caution (see Box 2).

Diagnosis		Data source	ICD-10-AM diagnosis code
Gestational age < 32 weeks*		Birth data	N/A
Birthweight < 1500g		Birth data	N/A
Death within 28 days of birth		Birth, hospital and mortality data	N/A
Birth trauma		Hospital data	P10.0, P10.1, P10.2, P10.3, P13.0, P13.2, P13.3, P14.0, P14.1
Cerebral conditions	
	Intraventricular haemorrhage	Hospital data	P52.1, P52.2
	Hypoxic-ischaemic encephalopathy	Hospital data	P91.2, P91.5, P91.81, P91.6
	Seizures	Hospital data	P90, R56
	Other cerebral diagnosis	Hospital data	I63
Respiratory conditions	
	Pneumonia	Hospital data	P23, J12, J13, J14, J15, J16, J17, J18
	Respiratory distress syndrome	Hospital data	P22.0
	Bronchopulmonary dysplasia	Hospital data	P27.1
	Other respiratory diagnosis	Hospital data	P28.0, P28.5
Sepsis/septicaemia		Hospital data	P36, A40, A41.5, A41.9, B95.1, B96.2
Necrotising enterocolitis		Hospital data	P77

Procedure		Data source	ACHI procedure code

Resuscitation or intubation recorded on birth record		Birth data	N/A
Transferred to an out of state facility within 24 hours		Hospital data	N/A
2 - 999 hours of mechanical ventilation†		Hospital data	N/A
Invasive ventilation procedure		Hospital data	92211-00, 13882-00, 13882-01, 13882-02, 13857-00, 13879-00, 22007, 22008, 90179
Non-invasive ventilation procedure‡		Hospital data	92209-00, 92209-01, 92209-02, 92038, 92039, 92040
Resuscitation procedure		Hospital data	92052, 92053, 92042-00, 90225
Arterial/central catheter procedure		Hospital data	38206, 38200-00, 38203-00, 13303-00, 34524-00, 34530-01, 13300-02, 13319-00, 13815
Transfusion of blood or blood products		Hospital data	13306-00, 13706-01, 13706-02, 13706-03, 13706-04, 92206-00
Intravenous fluid procedure		Hospital data	96199-00, 96199-01, 96199-03, 96199-04, 96199-05, 96199-06, 96199-07, 96199-08, 96199-09, 96199-10, 96199-19, 92181-00, 92182-00, 92183-00, 92184-00, 92185-00, 92188-00, 92192-00
Surgical procedures	
	Abdominal	Hospital data	31462-00, 30378-00, 30564-00, 30565-00, 30566-00, 30571-00, 30615-00, 30617-00, 32123-00, 43801-00, 43807-00, 43816-02, 43870-00, 43930-00, 43945-00, 43963-00, 30373, 30375, 30562, 30601, 43837, 43843, 43864, 43867, 43873, 43876, 43978
	Cardiac	Hospital data	38600-00, 90224-00, 96219-00, 96220-00, 387
	Cerebral	Hospital data	39640-00, 90032-00, 40100-00, 40103-00, 39015, 40003
	Thoracic	Hospital data	38403-00, 38803-00, 38409-00, 38806-00, 43852-00, 43900-00, 43915-00, 41881, 41883, 90180
	Urinary system	Hospital data	36624-01, 36516, 36537, 36564, 36579

From 2002 to 2014 there were 1,194,681 infants identified from the birth data, 99.3% with a matching hospital record. Of this cohort 5.44% (n = 65,024) had one or more diagnoses and/or procedures indicative of severe or medically significant neonatal morbidity. The morbidity rate increased over time, from 4.68% in 2002 to 6.77% in 2014. The most commonly flagged components of the morbidity indicator were intravenous infusion and respiratory system complications. The number of infants flagged by the indicator and by individual NAOI components is displayed in Table 2. The incidence of neonatal morbidity additionally split by gestational age is displayed in Supplementary Table 1.

**Table 2: Frequency, incidence per 100 births and influence for the neonatal adverse outcome indicator (NAOI) and components.** Influence is defined as the percentage reduction in the neonatal morbidity rate when this component is removed * Updated from previous published version of the NAOI [3]

				Percentage with NAOI component			Trend (P value, direction)
						
Component			N	Overall	2002	2014		Influence^
NAOI (overall)			65,024	5.44	4.68	6.77	<0.001, ↑	100.0%
Death		
	Died within 28 days of birth		2,483	0.2	0.3	0.2	<0.001, ↓	0.5%
Diagnosis		
	<32 weeks’ gestational age		10,803	0.9	0.9	0.9	0.068, --	0.5%
	<1500g birth weight		9,492	0.8	0.8	0.8	0.025, --	0.7%
	Birth trauma		1,074	0.1	0.1	0.1	0.512, --	1.2%
	Respiratory conditions	
		Respiratory distress syndrome	22,355	1.9	1.6	2.1	<0.001, ↑	6.3%
		Bronchopulmonary dysplasia	1,680	0.1	0.1	0.2	<0.001, ↑	<0.1%
		Pneumonia	1,110	0.1	0.1	0.1	<0.001, ↑	0.4%
		Other respiratory conditions	1,349	0.1	0.1	0.2	<0.001, ↑	0.3%
	Intraventricular haemorrhage		665	0.1	0.1	0.1	0.404, --	<0.1%
	Hypoxic-ischaemic encephalopathy		1,429	0.1	0.1	0.1	<0.001, ↑	0.1%
	Seizures		2,097	0.2	0.2	0.1	<0.001, ↓	0.6%
	Other cerebral conditions		131	<0.1	<0.1	<0.1	0.023, --	<0.1%
	Sepsis/septicaemia		5,590	0.5	0.6	0.5	<0.001, ↓	2.8%
	Necrotising enterocolitis		692	0.1	0.1	0.1	<0.001, ↓	<0.1%
Procedures		
	Resuscitation/intubation (birth record)		10,996	0.9	1.0	1.0	<0.001, ↑	4.1%
	≥2 hours mechanical ventilation*		12,224	1.0	1.2	1.0	<0.001, ↓	0.1%
	Transfer to an out-of-state facility		805	0.1	0.1	0.1	0.413, --	0.5%
	Non-invasive ventilation*		29,110	2.4	1.5	3.6	<0.001, ↑	12.7%
	Invasive ventilation*		14,224	1.2	1.4	1.1	<0.001, ↓	0.3%
	Resuscitation (hospital record)		641	0.1	<0.1	<0.1	0.776, --	0.1%
	Intravenous infusion*		31,309	2.6	2.1	3.9	<0.001, ↑	16.8%
	Transfusion of blood or blood products		6,057	0.5	0.5	0.5	0.996, --	0.4%
	Central catheter*		9,906	0.8	0.9	0.8	<0.001, ↓	0.8%
	Surgical procedures	
		Abdominal*	1,582	0.1	0.1	0.1	0.578, --	0.2%
		Cardiac*	1,093	0.1	0.1	0.1	0.135, --	<0.1%
		Cerebral*	192	<0.1	<0.1	<0.1	0.018, --	0.1%
		Thoracic*	1,241	0.1	0.1	0.1	0.041, --	<0.1%
		Urinary system	19	<0.1	<0.1	<0.1	0.323, --	<0.1%

Trend analysis (reported in Table 2) indicated an increased reported incidence of respiratory conditions and hypoxic-ischaemic encephalopathy over the study period. The incidence of seizures, sepsis/septicaemia, necrotising enterocolitis and death within the first 28 days decreased. For procedures, the incidence of non-invasive ventilation, resuscitation or intubation recorded on the birth record and intravenous infusion increased, while the incidence of central catheterisation, invasive mechanical ventilation and ≥ 2 hours of mechanical ventilation decreased. Intravenous infusion and diagnoses/procedures related to respiratory conditions had the largest influence on the number of infants identified as having neonatal morbidity (Table 2); interestingly, the influence of non-invasive ventilation increased over the study period (Supplementary Figure 1).

Gestational age-specific neonatal morbidity decreased with increasing gestational age, from 77.8% at 32 weeks to the nadir of 2.5% at 39 weeks, after which it increased to 3.8% at 42+ weeks’ gestation (Figure 1). All infants <32 weeks’ gestation (not shown in figure) were considered to have morbidity as defined by the NAOI.

**Figure 1: Rate of neonatal morbidity by gestational age at birth, from 32 weeks onwards. All infants of gestational ages <32 weeks are considered to have morbidity as defined by the neonatal adverse outcome indicator (NAOI).**
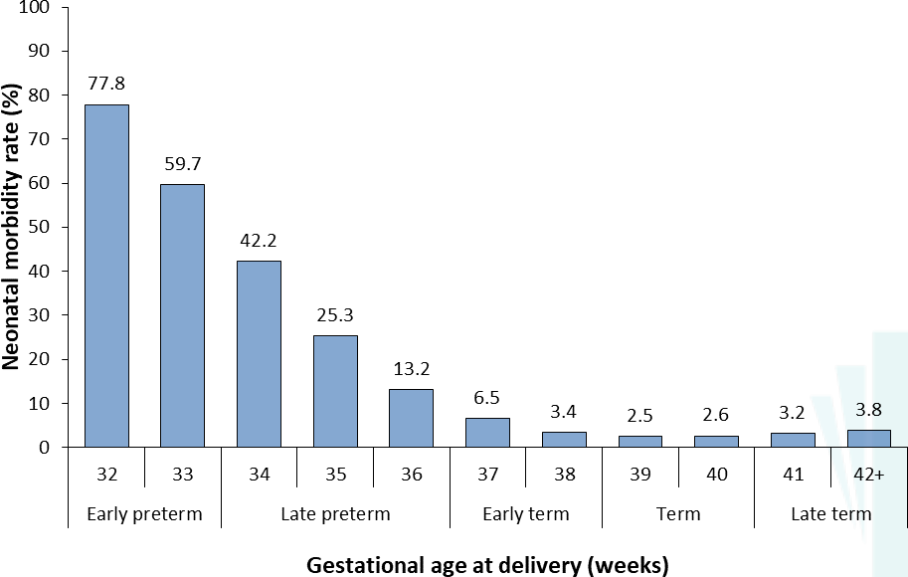


Population characteristics by morbidity are shown in Table 3. Women aged <20 years or 35+ years, nulliparous women, and smokers had increased odds of infants with neonatal morbidity. Morbidity was higher for private patients giving birth in a public hospital compared with private patients in private hospitals and public patients in public hospitals. For infants, increased odds of neonatal morbidity was seen for male gender, preterm birth, being large or small for gestational age, or being part of a multiple birth.

**Table 3: Frequency of infants with and without severe or medically significant morbidity, as identified by the neonatal adverse outcome indicator (NAOI) and association of neonatal morbidity with maternal and infant characteristics.** Only includes records with no missing data, N = 1,190,827 (99.7% of study cohort).

Maternal and infant characteristics		Infants identified by NAOI	Infants not identified by NAOI	Unadjusted odds ratio of neonatal morbidity
		N = 64,793 (%)	N = 1,126,034 (%)	(95% CI)
Maternal characteristic	
Maternal age	
	<20 years	4.1	3.5	1.21 (1.16 – 1.26)
	20 – 34 years	71.5	74.2	Reference
	35+ years	24.4	22.3	1.14 (1.12 – 1.16)
Nulliparous		49.8	42.1	1.36 (1.34 – 1.38)
Smoker		16.2	12.2	1.39 (1.36 – 1.42)
Patient status	
	Public patient in public hospital	76.9	73.9	Reference
	Private patient in public hospital	9.4	0.5	18.11 (17.44 – 18.80)
	Private patient in private hospital	13.8	25.6	0.52 (0.50 – 0.53)
Infant characteristic	
Mean birthweight		2704g	3417g	Not applicable
Male		58.1	51.1	1.33 (1.30 – 1.35)
Preterm (<37 weeks)		47.4	4.5	19.08 (18.75 – 19.43)
SGA		14.7	9.9	1.57 (1.54 – 1.61)
LGA		11.9	9.7	1.26 (1.23 – 1.29)
Multiple		13.3	2.4	6.35 (6.19 – 6.51)

For infants discharged home, those identified by the NAOI had double the rates of readmission to hospital in the first year of life, both for any readmission or for an overnight stay. This cohort also had 10 times the rate of death within the first year of life compared with infants not identified by the NAOI (Table 4).

**Table 4: Rates of hospital readmission or death among infants discharged home up until their first year, total and by components of the neonatal morbidity indicator (NAOI).** * Updated from previous published version of the NAOI [3]

Neonatal diagnosis or procedure			N	Rate of readmission (any) (%)	Rate of readmission (overnight) (%)	Rate of death (%)
All infants discharged home			1,187,950	18.4	10.6	0.2
	Infants flagged by NAOI		62,281	32.4	21.0	1.2
	Infants not flagged		1,125,669	17.6	10.0	0.1
Death		
	Died within 28 days of birth		275	37.5	11.3	-
Diagnosis		
	<32 weeks’ gestational age		9,496	48.5	33.0	1.0
	<1500g birth weight		8,233	49.3	33.2	1.3
	Birth trauma		1,051	22.7	13.8	0.2
	Respiratory conditions	
		Respiratory distress syndrome	21,355	37.9	25.1	0.8
		Bronchopulmonary dysplasia	1,574	57.9	40.4	1.9
		Pneumonia	1,035	35.7	23.0	0.7
		Other respiratory conditions	1,088	40.3	27.8	2.2
	Intraventricular haemorrhage		512	56.8	39.3	2.1
	Hypoxic-ischaemic encephalopathy		1,149	37.8	22.7	5.4
	Seizures		1,773	43.5	27.2	6.3
	Other cerebral conditions		120	46.7	22.5	3.3
	Sepsis/septicaemia		5,247	38.3	25.3	1.5
	Necrotising enterocolitis		577	52.9	39.2	3.6
Procedures		
	Resuscitation/intubation (birth record)		9,674	35.2	23.4	1.3
	≥2 hours mechanical ventilation*		10,750	47.7	33.6	2.5
	Transfer to an out-of-state facility		622	36.8	22.0	1.4
	Non-invasive ventilation*		28,456	35.3	23.3	0.8
	Invasive ventilation*		12,561	46.0	32.3	2.4
	Resuscitation (hospital record)		479	31.5	21.3	3.1
	Intravenous infusion*		30,235	36.1	24.0	1.0
	Transfusion of blood or blood products		5,262	53.1	37.2	2.8
	Central catheter*		8,967	46.5	31.7	2.0
	Surgical procedures	
		Abdominal*	1,482	56.9	43.9	2.9
		Cardiac*	993	67.0	52.5	7.7
		Cerebral*	182	72.0	63.2	6.0
		Thoracic*	1,075	48.1	32.7	2.4
		Urinary system	19	84.2	36.8	0.0

## Discussion

Accurately identifying infants with severe or medically significant neonatal morbidity in population-based datasets requires a tool that reflects current coding standards – this is particularly true for indicators with multiple components. We updated an existing neonatal morbidity indicator, the neonatal adverse outcomes indicator (NAOI) [3], to reflect changes in coding standards and diagnosis and procedure codes. The updated NAOI retains its ability to identify infants with neonatal morbidity and can be adapted to other health systems that use similar diagnostic and procedure codes, as has already been done in England (E-NAOI) [9], China [10], Canada [11], and Taiwan [12].

The proportion of infants flagged with neonatal morbidity was substantially higher for early gestational ages, consistent with previous estimates using this indicator and the adapted E-NAOI [3, 9]. Interestingly, while 37 weeks’ gestation is often considered term, the incidence of neonatal morbidity at 37–38 weeks (4.2%) was nearly double that at 39–40 weeks (2.5%), supporting the recommendation to redefine ‘full term’ as 39–40 weeks and 37–38 weeks as ‘early term’ [19]. Odds of neonatal morbidity also increased with known risk factors including size for gestational age [20], multiple births [21], and maternal characteristics such as smoking [22]. The increased rates of neonatal morbidity in public versus private hospitals were also expected given admission practices in Australia, with women whose pregnancies are predicted to require high level care for the neonate transferred to public tertiary facilities before birth [23]. Rates of hospital readmission (both any and overnight stays) and mortality within the first year of life were two and 10 times higher in flagged infants respectively, consistent with previous estimates [3]. The E-NAOI recorded a corresponding 2-fold and 15-fold increased risk [9].

The minimal change in the NAOI when individual code blocks are removed reflects the success of the indicator in identifying neonatal morbidity as morbid infants are likely to have multiple diagnoses and procedures recorded. However, some codes were more likely to occur in isolation, in particular non-invasive ventilation and intravenous infusion, the removal of which reduced the rate of neonatal morbidity by 12.7% and 16.8% respectively. Although neonatal morbidity continued to trend upwards from 2002 to 2014 when these two components were removed (data not shown), this trend was more pronounced using the complete indicator. However, removal of either of these procedure groups is problematic given both are appropriate indicators of medically significant neonatal morbidity.

Although we report that neonatal morbidity in the NSW population increased from 2002 to 2014, it is uncertain whether this change is driven by a genuine increase in incidence in the population or changes in practice or diagnostic criteria. In NSW the proportion of babies born between 37–41 weeks’ gestation has remained consistently above 90% [24, 25], however within this gestational age bracket there has been a left shift towards births at earlier gestations. In NSW the modal gestational age has decreased from 40 to 39 weeks, driven by an increase in the rate of planned births (i.e. induction or prelabour caesareans) before 40 weeks’ gestation [26]. This suggests the possibility that a decrease in gestational age may be increasing neonatal morbidity. However, during this time period population characteristics have also changed. In NSW, maternal and pregnancy risk factors such as higher maternal age and nulliparity have increased over the past decade, although this is in conjunction with reduced rates of maternal smoking and multiple births [24, 25].

This complexity is also evident within patient care. For instance, we know that at least some of the increase in non-invasive mechanical ventilation is due to a change in coding, moving from procedure-based to time-based coding as well as including previously unrecorded procedures such as high flow nasal cannula. However non-invasive ventilation of neonates in Australia has also increased, as a way to reduce rates of invasive mechanical ventilation (particularly intubation) and associated complications and length of stay in paediatric intensive care units [27]. Non-invasive ventilation is increasingly the first choice of support for respiratory distress syndrome [28]. In our cohort, we found invasive ventilation decreased from 1.4% to 1.1% over the study period, while non-invasive ventilation increased from 1.5% to 3.6%. This complex interplay of factors suggests that use of the NAOI in longitudinal studies should be undertaken cautiously, and investigation into the relative contributions of individual components should be part of the analysis process.

### Limitations

Optimal interpretation of the NAOI requires a good understanding of the influence of each of the individual components, along with knowledge of the historic changes in coding rules and neonatal medical practice. Classifications of neonatal severity may also differ by study question and research design, necessitating clear case definitions and research criteria. However, the definition of morbidity may also vary between clinicians, and between clinicians and facility administrative measures.

Ascertainment of individual components within the NAOI can vary - previous research in NSW which validated hospital ICD and ACHI coding against neonatal intensive care audit data found the sensitivity for diagnostic and procedure codes could range from 37% to 98% (although specificity was generally higher at >85%) [14]. However, as the neonatal morbidity indicator is designed to be used as an overall flag, rather than by individual components, these ascertainment issues are mitigated by the inclusion of the multiple components.

The ease of adaptation to different healthcare systems depends on data quality and equivalent coding practices, with an assessment of validity recommended. The adaptation of the NAOI to the English NHS resulted in the removal of blood transfusions and the addition of therapeutic hypothermia and bacterial meningitis, yet still produced consistent results [9].

Another limitation of this indicator is its binary nature – it cannot calculate ‘degrees’ of neonatal morbidity. Crude calculation of the number of flagged diagnoses or procedures, as a way of computing a degree of morbidity, may instead reflect administrative coding styles between hospitals. In addition, not all components are equally severe. This could be solved by weighting components by severity or the requirement for significant medical intervention, however the creation and validation of this measure would be labour intensive. 

## Conclusion

The updated composite indicator has maintained the concurrent and predictive validity of the original NAOI, and is a standardised, economic way to measure neonatal morbidity using population-based data. The composite nature of the indicator makes it robust against under-ascertainment, however individual components may shift over time due to practice or coding changes. In our study of the NSW population, this was evident for intravenous infusion and non-invasive ventilation procedures which both had significant changes to coding practice between 2002 and 2014. Rates of morbidity for individual components should be thoroughly investigated when examining the NAOI in longitudinal data to ensure any increases or decreases are attributed correctly.

## Acknowledgments

We thank the NSW Ministry of Health for access to the population health data and the Centre for Health Record Linkage for linking the datasets. We additionally thank Maiane Iboian and Lyndsey Harvey for their expert advice during the updating process.

This work was completed while Stephanie Todd was employed as a Trainee Biostatistician on the NSW Biostatistics Training Program, funded by the NSW Ministry of Health, and undertook this work whilst based at Women and Babies Research. Siranda Torvaldsen and Stephanie Todd were supported by funding from a NSW Ministry of Health Prevention Research Support Program grant.

## Statement of Ethics Approval

This study was approved by the New South Wales Population and Health Services Research Ethics Committee (2012/12/430). Routinely collected administrative data can be used for research with a waiver of participant consent following assessment of the study protocol. Only de-identified data is available for use by researchers and must be stored and analysed in a secure manner. 

## Supplementary Files

Supplementary Table 1 and Figure 1

## Supplementary Appendices

Supplementary Table 1. Incidence per 100 births for the neonatal adverse outcome indicator (NAOI) and components, by gestational age term groups.

Supplementary Figure 1. Sensitivity analysis of non-invasive ventilation procedures on the incidence of severe or medically significant neonatal morbidity, assessed by relevant codes from neonatal adverse outcome indicator (NAOI).

## Abbreviations

ACHI	Australian Classification of Health Interventions
ACS	Australian Coding Standards
E-NAOI	English neonatal adverse outcome indicator
ICD-10-AM	International Statistical Classification of Diseases and Related Health Problems, Tenth Revision, Australian Modification
NAOI	Neonatal adverse outcome indicator
NSW	New South Wales
