# Supplementary material for: A composite neonatal adverse outcome indicator using population-based data: an update
Source: Int J Popul Data Sci. 2020 Aug 12;5(1):1337. doi: 10.23889/ijpds.v5i1.1337 (PMC7893849; doi:10.23889/ijpds.v5i1.1337)

Supplementary Table 1. Incidence per 100 births for the neonatal adverse outcome indicator (NAOI) and components, by gestational age term groups.

Component	Incidence per 100 births					
	Overall	Early preterm (<34 weeks)	Late preterm (34 – 36 weeks)	Early Term (37 – 38 weeks)	Term (39 – 40 weeks)	Late term (41+ weeks)
NAOI (overall)	5.44	83.0	21.6	4.2	2.5	3.3
**Death**						
Died within 28 days of birth	0.2	6.1	0.5	0.1	0.1	0.1
**Diagnosis**						
<32 weeks' gestational age	0.9	50.3	0	0	0	0
<1500g birth weight	0.8	41.7	0.8	<0.1	<0.1	<0.1
Birth trauma	0.1	0.3	0.1	0.1	0.1	0.1
Respiratory conditions						
Respiratory distress syndrome	1.9	54.0	7.7	0.9	0.4	0.5
Bronchopulmonary dysplasia	0.1	7.6	<0.1	<0.1	<0.1	<0.1
Pneumonia	0.1	1.1	0.2	0.1	0.1	0.1
Other respiratory conditions	0.1	3.2	0.4	0.1	<0.1	<0.1
Intraventricular haemorrhage	0.1	2.8	<0.1	<0.1	<0.1	<0.1
Hypoxic-ischaemic encephalopathy	0.1	1.0	0.3	0.1	0.1	0.1
Seizures	0.2	1.2	0.4	0.2	0.1	0.2
Other cerebral conditions	<0.1	0.1	<0.1	<0.1	<0.1	<0.1
Sepsis/septicaemia	0.5	10.3	1.2	0.3	0.2	0.3
Necrotising enterocolitis	0.1	2.6	0.1	<0.1	<0.1	<0.1
**Procedures**						
Resuscitation/intubation (birth record)	0.9	21.1	1.5	0.5	0.4	0.7
≥2 hours mechanical ventilation*	1.0	30.4	2.9	0.6	0.3	0.3
Transfer to an out-of-state facility	0.1	0.9	0.3	<0.1	<0.1	<0.1
Non-invasive ventilation*	2.4	58.1	8.7	1.4	0.8	1.2
Invasive ventilation*	1.2	33.3	3.2	0.7	0.4	0.5
Resuscitation (hospital record)	0.1	0.5	0.1	0.1	<0.1	<0.1
Intravenous infusion*	2.6	51.9	11.5	1.9	0.9	1.0
Transfusion of blood or blood products	0.5	19.0	0.9	0.2	0.1	0.1
Central catheter*	0.8	26.2	2.3	0.4	0.2	0.2
Surgical procedures						
*Abdominal**	0.1	1.4	0.5	0.2	0.1	<0.1
*Cardiac**	0.1	1.1	0.2	0.1	0.1	<0.1
*Cerebral**	<0.1	0.2	<0.1	<0.1	<0.1	<0.1
*Thoracic**	0.1	1.9	0.4	0.1	<0.1	<0.1
Urinary system	<0.1	<0.1	<0.1	<0.1	<0.1	<0.1

* Updated from previous published version of the NAOI [3]

Supplementary Figure 1: Sensitivity analysis of non-invasive ventilation procedures on the incidence of severe or medically significant neonatal morbidity, assessed by relevant codes from neonatal adverse outcome indicator (NAOI).

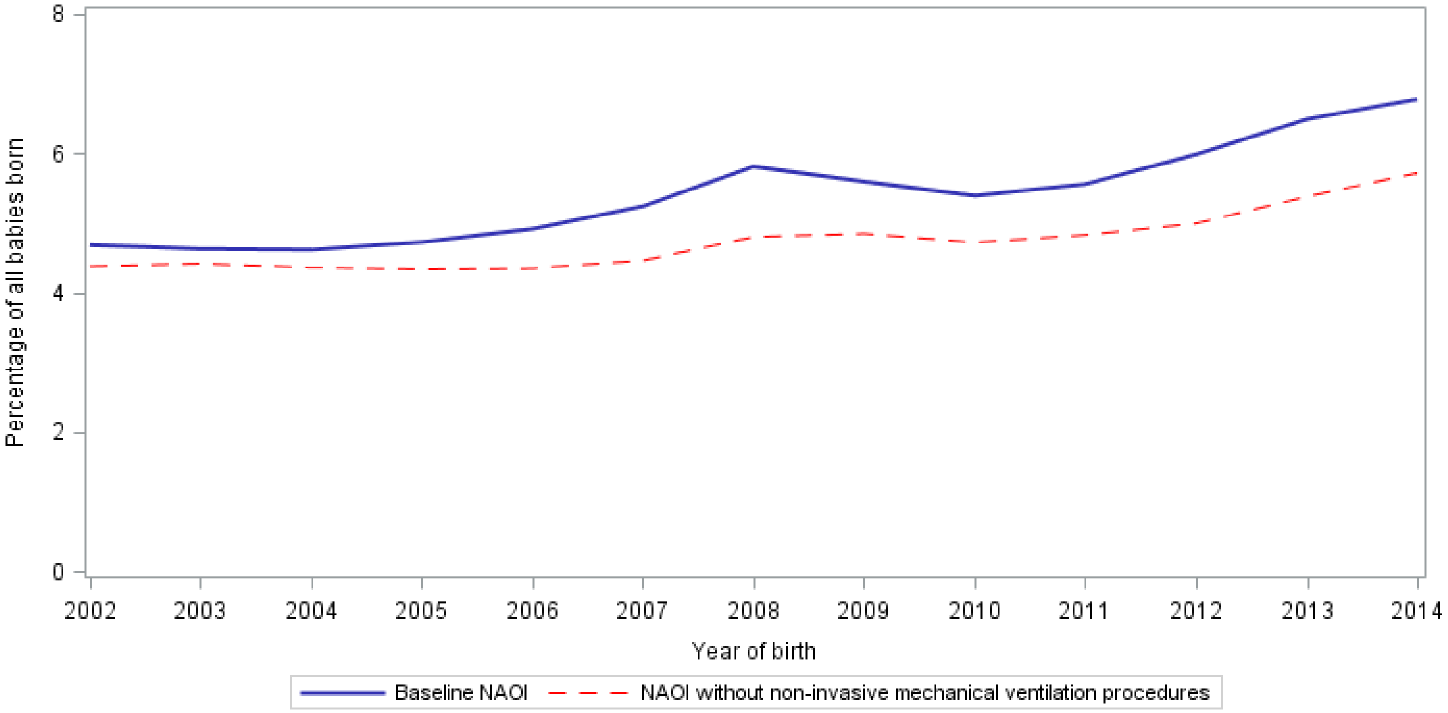

Supplement: Supplementary Table 1 and Figure 1 [file ijpds-05-1337-s001.pdf]
